# Physical and psychological needs of women undergoing hysterectomy: A literature review

**DOI:** 10.6026/973206300212231

**Published:** 2025-07-31

**Authors:** Nirmala A., Lilly Christopher, Shankar Shanmugam Rajendran, Jayabharthi B., Senthil Kumaran A., Revathi Ramasamy

**Affiliations:** 1 Department of Obstetrics & Gynaecological Nursing, Meenakshi Academy of Higher Education and Research (MAHER), Chennai, India; 2 Department of Child Health Nursing, College of Nursing, Madras Medical College, Chennai, India

**Keywords:** Emotional support, hormone replacement therapy, pain management, psychological well-being, recovery

## Abstract

The physical and psychological needs of women who have undergone hysterectomy, both immediately post-surgery and long-term are of interest. These include managing pain, addressing hormonal changes, emotional distress, and adjusting to a new body image. The importance of comprehensive care that combines physical recovery with psychological support is shown. Thus, the need for tailored healthcare strategies to enhance the overall well-being of women after hysterectomy is shown.

## Background:

Hysterectomy is the most common surgery performed on women globally, typically indicated for conditions like fibroids, endometriosis, uterine cancer, prolapse, or abnormal bleeding [1]. While the procedure is generally safe, it can significantly affect both physical and mental health. Post-surgery, women face numerous challenges, requiring comprehensive attention to physical and emotional recovery [2]. Pain management, especially in the abdominal and pelvic regions, is a primary concern, with many women needing multimodal interventions like medication and physical therapy [4]. Additionally, wound care and preventing infections are essential components of the recovery process, with women at risk for postoperative infections, particularly at the incision site [6]. The removal of ovaries can trigger hormonal changes, resulting in symptoms such as hot flashes, mood swings, and vaginal dryness, which can contribute to mental distress, often necessitating hormone replacement therapy (HRT) [7]. Furthermore, early postoperative restrictions, such as avoiding heavy lifting, are essential to prevent complications like blood clots and stress on the surgical site [8]. These physical limitations may be frustrating, as they hinder normal activities, which can affect emotional well-being, particularly in the absence of adequate support [9]. The psychological burden is often exacerbated for women undergoing hysterectomy due to cancer or infertility concerns. The loss of the ability to conceive can lead to feelings of grief and diminished self-worth, influencing both body image and mental health [10]. Counseling and emotional support plays a critical role in helping women navigate these feelings [11]. Sexual health concerns also emerge post-hysterectomy, with some women fearing diminished sexual satisfaction or functionality. Addressing these fears through education and physical therapy is vital to maintaining sexual well-being post-surgery [12]. Comprehensive pre-surgery education can alleviate anxiety and confusion by preparing women for the physical and emotional changes they will experience [13]. Thus, healthcare providers must address both physical and emotional needs, offering pain relief, hormone support, mental health care, and sexual health education to ensure a positive recovery and long-term well-being [14]. Therefore, it is of interest to report on the comprehensive care required to optimize recovery following hysterectomy.

## Review:

The initial search used the keywords and then the titles and abstracts of the retrieved papers were examined to determine their relevance. We read the entire texts of the documents that met the first set of requirements to determine if they were suitable for our purposes. This review of the literature looked at eight separate studies. They were in different parts of the world, employed various methods to study and examined diverse groups of people. These studies taught us a lot about the physical and mental needs of women who were having a hysterectomy. We reviewed the research data, focusing on the most significant impacts of hysterectomy on women's mental and physical health. People took out strategies to deal with pain, care for wounds, problems moving around and changes in hormones, needs for emotional support, sexual health issues and body image and how vital knowledge is for helping people get better. After that, the data were organized in a way that told a story about what the research found. There were two basic categories of data: those related to bodily needs and those related to mental demands. This plan helped us see how the physical and mental problems that come with hysterectomy are connected and how they affect the whole rehabilitation process. We applied standard procedures to assess the quality of each included study, ensuring the results were accurate and could be trusted. We utilized the Cochrane Collaboration's Risk of Bias Tool to determine the quality of the methodologies used in quantitative research. We examined aspects such as how the experiment was set up, how the randomization process worked and what happened to the data when it was missing.

We used the Critical Appraisal Skills Programme (CASP) Qualitative Checklist to assess the credibility and trustworthiness of the qualitative study results. Only research that satisfied a certain threshold is in the review. We employed a qualitative synthesis rather than a quantitative meta-analysis because the selected publications employed diverse study designs and methods for quantifying outcomes. Because research is conducted in various ways, this strategy enabled the review to encompass a range of diverse experiences and needs mentioned in the literature. The results were organized into groups based on topics such as mental and physical health. There were subcategories for each issue, like sexual health, body image, pain management and emotional support needs. After that, these themes were used to determine what type of full care women need after having their uterus removed. The systematic review has some issues. Because each study had a different design, it's hard to compare them directly. There may also be publication bias, which means that studies with significant or positive results are more likely to be published. The sample sizes for the study were also significantly variable; therefore, some of the conclusions may not be valid in other cases. Even though there are some issues with the exam, it gives us crucial information about the physical and mental needs of women who have undergone a hysterectomy. It also indicates where we need to conduct further investigation. This systematic literature analysis examines the results of eight studies to gain a deeper understanding of the physical and mental requirements of women who have undergone a hysterectomy. The results are aimed to make sure that women's psychological and physical health is both taken care of following surgery. This will make their treatment better.

Many women have hysterectomies to treat gynecological disorders. This treatment has a big influence on their bodies and minds. The therapy is frequently necessary for medical reasons, but the time it takes to recuperate can be quite challenging. Women may endure pain after surgery, have hormonal problems and need long-term care, especially if their ovaries are also removed. After an oophorectomy, people may have to do less, be in pain and require hormone replacement therapy (HRT) to help them heal. These changes can make a woman's life much worse by giving her symptoms like hot flashes, vaginal dryness and tiredness [15]. This literature review, presented in Table 1 (see PDF), synthesizes eight key studies published between 2000 and 2025, examining the physical and psychological needs of women undergoing hysterectomies. These studies offer valuable insights into the postoperative recovery process, examining both physical recovery and psychological well-being, which are crucial components of the overall healing process.

## Discussion:

This review of the literature looked at a variety of studies that give important information regarding the physical and mental needs of women who have had a hysterectomy. The study reveals that individuals undergo significant changes, including improvements in their physical condition and the development of emotional management skills. We need to know a lot more about what women need after surgery so that we can better assist them. Erickson *et al.* (2022) [16] indicate that these alterations to the body may persist for a prolonged period, especially when perform concurrently with oophorectomy, the removal of the ovaries. Hormones can have a big effect on women's health. They can make you tired, give you hot flashes and dry up your vagina. Hormone replacement therapy (HRT) can assist with some of these problems, but it takes a long time for the body to heal after surgery. These results demonstrate that patients require ongoing medical care tailored to their individual needs, which includes maintaining stable hormone levels and improving their overall well-being. A lot of ladies feel a lot of emotions after having a hysterectomy. Many people reported feeling sad, alone and uncertain about their identity. This is not especially true for women who had the surgery because they couldn't get pregnant nor had cancer [17]. All of the studies that were looked at show that having a hysterectomy might not be good for a person's mental health. All of them believe that having counseling and emotional support is extremely important. Anna and Anjana's (2025) [18] study also shows that many women feel melancholy after having a hysterectomy. This emotional pain shows how important it is for women who are having mental health problems after the procedure to get care. What people think about femininity and fertility, which are commonly linked to having a uterus, can make the emotional burden much heavier. As emphasized by Mahardika *et al.* (2021) [19], the post-hysterectomy recovery process involves significant physical and emotional challenges, with women often experiencing both physiological changes and psychological distress. Their review underscores the importance of addressing these needs through individualized care plans that incorporate not only medical management to stabilize hormone levels and promote physical recovery but also psychological support to help women navigate the emotional impact of the surgery. The authors advocate for ongoing counseling and peer support, recognizing that mental health and emotional well-being are integral to the overall recovery process, especially in the context of changes in femininity and fertility perceptions. The results demonstrate that patients can experience improved mental well-being if they are informed about the operation in advance and then discuss it with someone afterward. Talking to other individuals who have been through the same issue can also be helpful at support groups.

## Conclusion:

It is important to meet the physical and mental requirements of women who are having a hysterectomy. These women need more than just medical care after surgery to improve livelihood. They also require help with their mental and emotional health. There is a need to heal both your body and your mind. Thus, a comprehensive approach to caring for women will help them manage the issues that arise after a hysterectomy.
